# The impact of obesity in hospitalized patients with COVID-19: a retrospective cohort study

**DOI:** 10.1186/s13098-023-01246-z

**Published:** 2024-01-19

**Authors:** Fábio Alfano Carra, Maria Edna de Melo, Matheo A. M. Stumpf, Cintia Cercato, Ariana E. Fernandes, Marcio C. Mancini, EPICCoV Study Group, Adriana Hirota, Alberto Kendy Kanasiro, Alessandra Crescenzi, Amanda Coelho Fernandes, Anna Miethke-Morais, Arthur Petrillo Bellintani, Artur Ribeiro Canasiro, Bárbara Vieira Carneiro, Beatriz Keiko Zanbon, Bernardo Pinheiro, Senna Nogueira Batista, Bianca Ruiz Nicolao, Bruno Adler Maccagnan Pinheiro Besen, Bruno Biselli, Bruno Rocha De Macedo, Caio Machado Gomes De Toledo, Carlos Roberto Ribeiro De Carvalho, Caroline Gomes Mol, Cassio Stipanich, Caue Gasparotto Bueno, Cibele Garzillo, Clarice Tanaka, Daniel Neves Forte, Daniel Joelsons, Daniele Robira, Eduardo Leite Vieira Costa, Elson Mendes Da Silva Júnior, Fabiane Aliotti Regalio, Gabriela Cardoso Segura, Giulia Sefrin Louro, Gustavo Brasil Marcelino, Yeh-Li Ho, Isabela Argollo Ferreira, Jeison Oliveira Gois, Joao Manoel Da Silva-Jr, Jose Otto Reusing Junior, Julia Fray Ribeiro, Juliana Carvalho Ferreira, Karine Vusberg Galleti, Katia Regina Silva, Larissa Padrao Isensee, Larissa Santos Oliveira, Leandro Utino Taniguchi, Leila Suemi Letaif, Lígia Trombetta Lima, Lucas Yongsoo Park, Lucas Chaves Netto, Luciana Cassimiro Nobrega, Luciana Bertocco Paiva Haddad, Ludhmila Abrahao Hajjar, Luiz Marcelo Sa Malbouisson, Manuela Cristina Adsuara Pandolfi, Marcelo Park, Maria José Carvalho Carmona, Maria Castilho Prandini H. Andrade, Mariana Moreira Santos, Matheus Pereira Bateloche, Mayra Akimi Suiama, Mayron Faria de Oliveira, Mayson Laercio Sousa, Michelle Louvaes Garcia, Natassja Huemer, Pedro Vitale Mendes, Paulo Ricardo Gessolo Lins, Pedro Gaspar Dos Santos, Pedro Ferreira Paiva Moreira, Renata Mello Guazzelli, Renato Batista Dos Reis, Renato Daltro-Oliveira, Roberta Muriel Longo Roepke, Rodolpho Augusto Moura Pedro, Rodrigo Kondo, Samia Zahi Rached, Sergio Roberto Silveira Da Fonseca, Thais Sousa Borges, Thalissa Ferreira, Vilson Cobello Junior, Vivian Vieira Tenório Sales, Willaby Serafim Cassa Ferreira

**Affiliations:** 1 Unidade de Obesidade, Divisão de Endocrinologia e Metabologia, Hospital das Clínicas da Faculdade de Medicina da Universidade São Paulo, R. Dr. Ovídio Pires de Campos, 225 - Cerqueira César, São Paulo, SP 05403-010 Brasil; 2 Laboratório de Carboidratos e Radioimuniensaio (LIM-18), Faculdade de Medicina da Universidade São Paulo, São Paulo, Brasil

**Keywords:** COVID-19, Obesity, Mortality, Mechanical ventilation, Critical care

## Abstract

**Background:**

Obesity is believed to be a risk factor for COVID-19 and unfavorable outcomes, although data on this remains to be better elucidated.

**Objective:**

To evaluate the impact of obesity on the endpoints of patients hospitalized due to SARS-CoV-2.

**Methods:**

This retrospective cohort study evaluated patients hospitalized at a tertiary hospital (Hospital das Clínicas da Faculdade de Medicina da USP) from March to December 2020. Only patients positive for COVID-19 (real-time PCR or serology) were included. Data were collected from medical records and included clinical and demographic information, weight and height, SAPS-3 score, comorbidities, and patient-centered outcomes (mortality, and need for mechanical ventilation, renal replacement therapy, or vasoactive drugs). Patients were divided into categories according to their BMI (underweight, eutrophic, overweight and obesity) for comparison porpoise.

**Results:**

A total of 2547 patients were included. The mean age was 60.3 years, 56.2% were men, 65.2% were white and the mean BMI was 28.1 kg/m^2^. SAPS-3 score was a risk factor for all patient-centered outcomes (HR 1.032 for mortality, OR 1.03 for dialysis, OR 1.07 for vasoactive drug use, and OR 1.08 for intubation, p < 0.05). Male sex increased the risk of death (HR 1.175, p = 0.027) and dialysis (OR 1.64, p < 0.001), and underweight was protective for vasoactive drug use (OR 0.45, p = 0.027) and intubation (OR 0.31, p < 0.003).

**Conclusion:**

Obesity itself was not an independent factor for worse patient-centered outcomes. Critical clinical state (indirectly evaluated by SAPS-3) appears to be the most important variable related to hard outcomes in patients infected with COVID-19.

## Highlights

- Obesity is present in up to 30% of patients hospitalized with COVID-19
- Obesity itself is not related to key endpoints such as mortality and need for dialysis, orotracheal intubation, or vasoactive drug
- SAPS-3 is the best predictor for worse outcomes in SARS-CoV-2 infection

## Introduction

During 2019, the world suffered a pandemic due to SARS-CoV-2 (Severe Acute Respiratory Syndrome Coronavirus 2) infection. The COVID-19 (Corona Virus Disease 2019) is believed to be responsible for almost 16 million deaths worldwide [1].

People with obesity are a risk population for this disease. In a cohort with almost 6000 hospitalized patients with COVID-19, half of them had obesity [2]. Obesity appeared to be also a risk factor for more severe disease and, consequently, increased mortality [3]. The mechanism behind this is probably related to premature immunosenescence, delayed hyperinflammation, and cytokine storm. In fact, adipocytes often secret more leptin, IL-6, TNF-alpha and INF-1, impairing residual immunological response [4].

In addition, concerning the pathophysiology, the virus uses the angiotensin-converting enzyme 2 receptor to infect the cell and replicate [5]. It is well-known that the adipose tissue may be vulnerable to more infection due to more expression of this receptor. People with obesity also have decreased chest-wall elastance, which leads to lower total respiratory compliance with a reduction of expiratory reserve volume and a higher susceptibility to infection. There is an impairment in total lung capacity and an increase in airway resistance as well as ventilation-perfusion mismatch [6].

Currently, COVID-19 treatment is based on supportive measures. In early stages of the pandemic, with the results of the RECOVERY trial, dexamethasone was a pillar treatment for hospitalized patients on invasive mechanical ventilation or oxygen supplementation alone, resulting in a lower 28-day mortality rate [7]. More recently, other drugs were studied for managing COVID-19 (such as remdesivir and tocilizumab) [8], but with an overall lower cost–benefit ratio than dexamethasone [9].

Currently, there are conflicting data on the role of obesity as an independent factor for mortality in hospitalized COVID-19 [10–12]. The disease severity itself (evaluated by desaturation, reduced level of consciousness, elevated creatinine) seemed to be the most relevant factor in predicting survival rate [13]. Therefore, the role of obesity alone as a factor for worse outcomes in patients with COVID-19 deserves better elucidation.

The aim of this article is to evaluate how obesity influenced the evolution and outcomes of hospitalized patients with COVID-19 in a single tertiary hospital center.

Methods.

This was a retrospective cohort study in a tertiary hospital center (Hospital das Clínicas da Faculdade de Medicina da USP), a reference for treatment care during the COVID pandemic, receiving complex cases with moderate-to-severe SARS-CoV-2 infection.

**Fig. 1:**
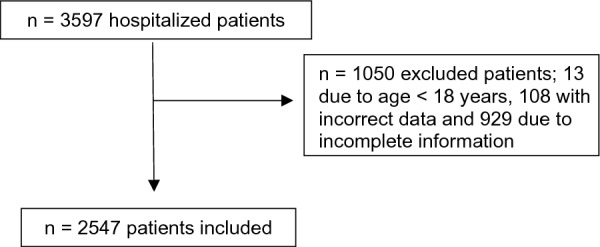
Flowchart of included patients

Data were collected from medical records during March 2020 to December 2020. The inclusion criteria were the presence of COVID-19 (symptoms alongside a confirmatory test, such as serology or real-time polymerase chain reaction), more than 18 years of old, moderate-to-severe disease that needed hospitalization, and presence of weight and height at admission. Patients with less than 18 years, incomplete data (without weight and height) or incorrect data (typeset errors) were excluded (Fig. 1).

For comparison porpoise, patients were stratified by body mass index (BMI, in kg/m^2^) in underweight (BMI < 18.5), eutrophic (18.6–24.9), overweight (25–29.9), class I obesity (30–34.9), class II obesity (35–39.9) and class III obesity (> 40). The primary endpoint was all-cause mortality. Secondary outcomes evaluated were the use of vasoactive drugs and the need for dialysis or mechanical ventilation. The SAPS-3 Score [14] was also calculated for staging severity, being overall a good predictor of mortality. Classically the variables computed for this score are age, PaO2, arterial pH, heart rate, creatinine, Glasgow coma scale, total bilirubin, leukocytes, systolic blood pressure, vasoactive drug, among others.

Descriptive and comparative analyses were presented. Data were summarized as mean ± standard deviation for continuous variables and as counts and percentages for categorical variables. For group comparisons, the chi-square test was used for categorical variables. Comparative analysis of the quantitative variables was presented using the analysis of variance (ANOVA) with Bonferroni correction and the likelihood ratio test. For the primary outcome, mortality was analyzed as a time-to-event measurement, with hazard ratio (HR) calculated by Cox regression. First, a univariate logistic regression was made and the variables that presented p < 0.10 were included in the multivariable Cox regression, as a stepwise selection of covariates. Odds ratios (OR) with 95% confidence intervals (CI) were calculated in a multivariable logistic regression test for the determination of risk factors for the secondary endpoints. The stepwise approach used in Cox regression was also used for logistic regression.

The variables used in the multivariable Cox regression for the primary endpoint were BMI, gender, and SAPS-3. The variables used in the multivariable logistic regression for the secondary endpoints for dialysis required were gender, and SAPS-3, and for vasoactive drugs use and need for mechanical ventilation were underweight, gender and SAPS-3.

The p-value < 0.05 was considered statistically significant. Statistical analysis of the data was performed using Statistical Package for Social Science (SPSS) version 17.0. The study was approved by the local Ethics Committee.

## Results

A total of 2547 patients were included. The mean age was 60.3 ± 15.7 years, 1431 (56.2%) were men and 603 (65.2%) were white. The most common comorbidity was hypertension in 1530 (60.1%) patients, followed by type 2 diabetes in 38.7% and smoking in 23.1%. The mean BMI was 28.1 ± 7.5 kg/m^2^.

Table 1 shows baseline variables in the groups stratified by BMI. Class III obesituently observed at younger ages and in women. This population also had more hypertension, asthma, and diabetes, but a lower SAPS-3 score.

**Table 1:** Demographic and clinical variables with comorbidities distribution according to BMI level

Variable	Total (n = 2547)	Underweight (n = 75, 2.9%)	Eutrophic (n = 847, 33.3%)	Overweight (n = 878, 34.5%)	Class I obesity (n = 396, 15.5%)	Class II obesity (n = 171, 6.7%)	Class III obesity (n = 180, 7.1%)	p
Age (years), mean (± SD)	60.3 (15.7)	60.6 (22.0)	60.7 (15.7)	63.7 (14.8)	58.16 (14.5)	55.2 (14.4)	50.9 (14.9)	** < 0.001**^**(1)**^
Male sex, no. (%)	1431 (56.2)	44 (58.7)	535 (63.2)	516 (58.8)	197 (49.8)	66 (28.6)	73 (40.6)	** < 0.001**^**(2)**^
Race, no. (%)								0.075^(2)^
White	1603 (65.2)	44 (63.8)	535 (65.6)	574 (68.0)	236 (60.5)	107 (64.1)	107 (62.8)	
Black	183 (7.5)	8 (11.6)	62 (7.6)	54 (6.4)	28 (7.2)	14 (8.4)	17 (9.9)	
Brown	643 (26.2)	14 (20.3)	207 (25.4)	207 (24.5)	123 (31.5)	45 (27.0)	47 (27.5)	
Asian	28 (1.1)	3 (4.4)	12 (1.5)	9 (1.1)	3 (0.8)	1 (0.6)	0 (0.0)	
SAPS-3, mean (± SD)	65.3 (16.5)	61.6 (13.7)	65.0 (15.5)	67.6 (16.6)	64.4 (17.7)	63.2 (17.4)	59.9 (16.9)	** < 0.001**^**(1)**^
Comorbidities, no. (%)								
Previous cardiovascular disease	529 (20.8)	14 (18.7)	182 (21.5)	194 (22.1)	82 (20.7)	29 (17.0)	28 (15.8)	0.348^(2)^
Hypertension	1530 (60.1)	29 (38.7)	459 (54.3)	552 (62.9)	253 (63.9)	118 (69.0)	119 (66.1)	** < 0.001**^**(2)**^
Arrhythmia	134 (6.7)	3 (5.5)	48 (7.3)	55 (7.4)	16 (5.4)	8 (6.5)	4 (3.7)	0.654^(2)^
COPD	186 (7.3)	8 (10.7)	65 (7.7)	75 (8.6)	18 (4.6)	8 (4.7)	12 (6.7)	0.083^(2)^
Asthma	104 (4.1)	1 (1.3)	31 (3.7)	21 (2.4)	20 (5.1)	11 (6.4)	20 (11.1)	** < 0.001**^**(2)**^
Dialysis CKD	87 (3.4)	7 (9.3)	40 (4.7)	25 (2.9)	9 (2.3)	3 (1.8)	3 (1.7)	**0.003**^**(2)**^
Non-dialysis CKD	245 (9.6)	10 (13.3)	92 (10.9)	85 (9.7)	41 (10.4)	11 (6.4)	6 (3.3)	**0.022**^**(2)**^
Liver disease	74 (2.9)	0 (0.0)	32 (3.8)	23 (2.6)	10 (2.5)	6 (3.5)	3 (1.7)	0.287^(2)^
Previous stroke or TIA	207 (8.1)	7 (9.3)	82 (9.7)	84 (9.6)	24 (6.1)	5 (2.9)	5 (2.8)	** < 0.001**^**(2)**^
Transplant	82 (20.3)	4 (25.0)	34 (24.8)	24 (17.5)	12 (20.0)	6 (20.7)	2 (8.0)	0.423^(2)^
Type 2 diabetes	986 (38.7)	14 (18.7)	287 (33.9)	354 (40.4)	175 (44.2)	79 (46.2)	77 (42.8)	** < 0.001**^**(2)**^
Dyslipidemia	172 (37.2)	3 (25.0)	42 (30.4)	74 (43.0)	28 (39.4)	15 (38.5)	10 (33.3)	0.275^(2)^
History of venous thrombosis	77 (20.2)	4 (30.8)	26 (20.5)	25 (19.8)	12 (19.7)	5 (17.9)	5 (18.5)	0.957^(2)^
Solid cancer	285 (11.8)	21 (31.3)	116 (14.0)	92 (11.0)	34 (9.2)	14 (8.6)	8 (5.0)	** < 0.001**^**(2)**^
Leukemia, lymphoma or myeloma	87 (4.4)	3 (5.6)	33 (5.0)	37 (5.0)	4 (1.4)	6 (4.9)	4 (3.7)	0.157^(2)^
Congenital immunodeficiency	43 (6.6)	0 (0.0)	17 (8.6)	14 (6.1)	7 (6.5)	4 (7.7)	1 (1.9)	0.308^(3)^
HIV	35 (1.4)	5 (6.7)	14 (1.7)	7 (0.8)	4 (1.0)	4 (2.3)	1 (0.6)	**0.018**^**(3)**^
Current smoking	184 (7.2)	4 (5.3)	68 (8.1)	62 (7.1)	27 (6.8)	8 (4.7)	15 (8.4)	0.647^(2)^
Former smoking	587 (23.1)	17 (22.7)	193 (22.9)	220 (25.1)	83 (21.1)	41 (24.1)	33 (18.3)	0.375^(2)^

*COPD* chronic obstructive pulmonary disease, *CKD* chronic kidney disease, *TIA* transient ischemic attack, *HIV* human immunodeficiency virus. (1) ANOVA; (2) chi-squared test; (3) likelihood ratio test

Bold indicates the variables with statistical significance (*p* < 0.05)

Concerning the primary outcome, multivariate Cox analysis determined only the male sex and SAPS-3 score being significantly associated with the risk of death (Table 2). Men had almost 20% more chance of dying than women and for each point in SAPS-3, the mortality likelihood increased by 3%.

**Table 2:** Cox regression for mortality outcome

Variables		All-cause mortality	
		Univariate model	Multivariate model
Male sex	HR	1.135	1.175
	95% CI	(0.981; 1.312)	(1.019; 1.356)
	p	0.088	**0.027**
SAPS-3	HR	1.031	1.032
	95% CI	(1.026; 1.035)	(1.027; 1.036)
	p	** < 0.001**	** < 0.001**
Underweight	HR	0.894	–
	95% CI	(0.512; 1.561)	–
	p	0.693	–
Eutrophic (reference)	HR	1,000	1.000
	95% CI	–	–
	p	–	–
Overweight	HR	1.139	–
	95% CI	(0.971; 1.335)	–
	p	0.110	–
Class I obesity	HR	0.889	–
	95% CI	(0.711; 1.113)	–
	p	0.305	–
Class II obesity	HR	0.834	–
	95% CI	(0.597; 1.164)	–
	p	0.286	–
Class III obesity	HR	0.727	–
	95% CI	(0.522; 1.012)	–
	p	0.059	–

Bold indicates the variables with statistical significance (*p* < 0.05)

The evaluation of patient-centered secondary outcomes (dialysis, vasoactive drug, and mechanical ventilation) showed that a higher SAPS-3 score is a risk factor for all of them (Table 3). Male sex was associated with the intrahospital need for dialysis. Interestingly, underweight was found to be protective against vasoactive drugs and mechanical ventilation.

**Table 3:** Logistic regression analysis for secondary main outcomes

Variables		Dialysis required		Vasoactive drug use		Need of mechanical ventilation	
		Univariate model	Mutivariate model	Univariate model	Mutivariate model	Univariate model	Mutivariate model
Male sex	OR	1.689	1.642	0.905	–	0.893	–
	95% CI	(1.354; 2.108)	(1.322; 2.040)	(0.685; 1.195)	–	(0.671; 1.189)	–
	p	** < 0.001**	** < 0,001**	0.483	–	0.439	–
SAPS-3	OR	1.031	1.031	1.079	1.078	1.088	1.088
	95% CI	(1.025; 1.038)	(1.024; 1.038)	(1.067; 1.090)	(1.067; 1.090)	(1.076; 1.101)	(1.076; 1.101)
	p	** < 0.001**	** < 0.001**	** < 0.001**	** < 0.001**	** < 0.001**	** < 0.001**
Underweight	OR	0.921	–	0.412	0.415	0.313	0.316
	95% CI	(0.426; 1.993)	–	(0.189; 0.897)	(0.191; 0.903)	(0.144; 0.677)	(0.146; 0.683)
	p	0.835	–	**0.026**	**0.027**	**0.003**	**0.003**
Eutrophic (reference)	OR	1.000	1.000	1.000	1.000	1.000	1.000
	95% CI	–	–	–	–	–	–
	p	–	–	–	–	–	–
Overweight	OR	0.963	–	1.133	–	1.168	–
	95% CI	(0.749; 1.237)	–	(0.816; 1.574)	–	(0.836; 1.632)	–
	p	0.766	–	0.457	–	0.362	–
Class I obesity	OR	1.323	–	1.083	–	1.335	–
	95% CI	(0.958; 1.828)	–	(0.713; 1.647)	–	(0.862; 2.068)	–
	p	0.089	–	0.707	–	0.196	–
Class II obesity	OR	0.918	–	1.070	–	1.439	–
	95% CI	(0.561; 1.501)	–	(0.589; 1.945)	–	(0.763; 2.715)	–
	p	0.733	–	0.823	–	0.261	–
Class III obesity	OR	1.366	–	0.799	–	1.202	–
	95% CI	(0.892; 2.093)	–	(0.485; 1.316)	–	(0.708; 2.039)	–
	p	0.152	–	0.378	–	0.496	–

Bold indicates the variables with statistical significance (*p* < 0.05)

## Discussion

Classically critical patients are at greater risk of unfavorable endpoints. Several trials have shown directly (by SatO2, acute renal failure, coma) or indirectly (by scores) higher mortality rates in more severe patients. The HOPE-COVID-19-Registry [13] evaluated retrospectively more than 3000 patients with COVID-19. The Cox multivariate analysis for mortality determined age ≥ 70 years, ICU admission, SpO2 < 92%, Glasgow Coma Scale < 15, connective tissue disease, and elevated creatinine as independent predictors for mortality. BMI, on the other hand, did not affect the mortality rate.

Some authors have proposed an obesity paradox during COVID-19. People with obesity would have a greater chance for ICU admission [10, 15] and mechanical ventilation [16], but not for mortality. Others, on the other hand, have shown that obesity is a neutral factor for these outcomes [11, 12].

An interesting study [17] evaluated the relationship between the mortality of COVID-19 and obesity classes according to BMI, visceral adipose tissue and muscle area. Patients > 70 years, with low muscle area (< 92 cm^2^) and BMI < 30 had a lower survival rate (HR 3.89–9.66, p < 0.0006). Patients with obesity and muscle area > 92 cm^2^ had a higher survival rate and obesity as an isolated parameter was not associated with mortality. This shows that the evaluation of BMI alone is very complex. Muscle area was significantly reduced in critical patients compared to noncritical patients, indirectly characterizing a more severe disease.

In contrast to the previous data, a recent Cochrane Review [18] identified those with class III obesity to be at increased odds for mortality (OR 1.67, 95% CI 1.39–2.00) compared to normal BMI or patients without obesity. Another study [19], set in Brazil (state of Espírito Santo), showed a twofold risk for death in people with obesity. It is unclear if these studies included adjustments in mortality rate by the baseline severity of the disease, so controversy remains. It is fair to imagine that patients with BMI > 40 have more comorbidities and a higher risk for severe disease, the latter being the true responsible for increased mortality. In fact, eutrophic people with multiple organ dysfunction have a higher chance of unfavorable outcomes than a person with obesity alone. In our study, people with BMI > 40 had significantly lower SAPS-3 score, which partially explained why more severe obesity itself was not correlated with mortality. Some hypothesis justifies the lower SAPS-3 in this group: by chance; a lower threshold for hospitalization due to severe obesity; earlier mortality or difficulty in accessing primary or secondary medical care, affecting the referral rate to a tertiary hospital center.

The Cochrane Review [18] also observed, for mechanical ventilation, increasing odds with higher classes of obesity in comparison to normal BMI or patients without obesity (class I: OR 1.38, 95% CI 1.20–1.59; class II: OR 1.67, 95% CI 1.42–1.96; class III: OR 2.17, 95% CI 1.59–2.97). In our study, underweight was protective against intubation. Interestingly, the already cited HOPE-COVID-19-Registry [13] also demonstrated that BMI < 25 was an independent factor for a lower rate of respiratory insufficiency.

Another meta-analysis [20] found that obesity prevalence rates were 32% in hospitalized patients, 43% in patients needing invasive mechanical ventilation, and 33% in those who died. Obesity was associated with a higher risk for hospitalization, ICU admission, and intubation requirement, but no increase in risk of death. Of note, the prevalence of pooled obesity (class I, II and III) in our study was similar, about 30%. As a matter of comparison, the recent VIGITEL inquiry revealed a 24.3% rate of BMI > 30 kg/m^2^ in the city of São Paulo [21]. This indirectly shows that people with obesity have an increased risk for hospitalization [19], since our rate in hospitalized patients was 6% higher than the observed in an overall healthy population.

The finding that men had higher mortality rates and dialysis appears to be something established and consolidated in the literature [19, 22, 23]. There are several mechanisms for worse COVID-19-related outcomes in the male sex: woman have a higher rate of vaccination and seek medical care more often and at early stages of the disease [24], inherent immunological differences (X chromosome has the largest number of genes related to the integrity of immune system) [23], and the higher testosterone levels (which facilitates SARS-CoV-2 entry via angiotensin-converting enzyme 2 expressed on cell surfaces) [25]. However, it should be highlighted that at the time of our study, vaccines were not yet universally available.

This study had some limitations. The data was retrospective and being a reference hospital for COVID-19 treatment, more severe cases were evaluated. Another limitation was the restriction to hospitalized patients. However, the high sample included should be noticed as a strength.

## Conclusion

Clinical critical condition (as evaluated by SAPS-3 score) is the best predictor for undesirable outcomes in COVID-19, namely mortality, need for dialysis, mechanical ventilation, or vasoactive drug. BMI itself was not an independent predictor of mortality; however, underweight patients might have lower intubation rates and vasoactive drug use than the control population.

More studies are needed to correctly assess the burden of obesity in COVID-19. Ideally, controlled trials with cases at the same severity levels but with different BMI should be conducted, trying to evaluate the isolated impact of obesity on clinically relevant outcomes.

## Data Availability

Our institution has an institution-wide data management plan for COVID-19 datasets which includes making anonymized data publicly available to contribute to nationwide and international registries of COVID-19 patients according to a pre-defined schedule.
